# Shoeshine maneuver for cystic duct dissection: a simple technique to make Calot-triangle dissection smooth

**DOI:** 10.1590/acb395224

**Published:** 2024-08-05

**Authors:** Marcelo Augusto Fontenelle Ribeiro, Roberto Rizzi, Sariya Khan, Maryam Makki, Shahin Mohseni

**Affiliations:** 1 University of Maryland – School of Medicine – R Adams Cowley Shock Trauma Center –Baltimore (MD) – United States.; 2 Hospital São Luiz – Department of Surgery – São Paulo (SP) – Brazil.; 3 Batterjee Medical College – Jeddah – Saudi Arabia.; 4 Orebro University – School of Medical Sciences – Department of Surgery – Orebro – Sweden.

**Keywords:** Cholecystectomy, Laparoscopic, Common Bile Duct, Surgical Procedures, Operative, Wounds and Injuries, Iatrogenic Disease

## Abstract

**Purpose:**

Laparoscopic cholecystectomy, introduced in 1985 by Prof. Dr. Erich Mühe, has become the gold standard for treating chronic symptomatic calculous cholecystopathy and acute cholecystitis, with an estimated 750,000 procedures performed annually in the United States of America. The risk of iatrogenic bile duct injury persists, ranging from 0.2 to 1.3%. Risk factors include male gender, obesity, acute cholecystitis, previous hepatobiliary surgery, and anatomical variations in Calot’s triangle. Strategies to mitigate bile duct injury include the Critical View of Safety and fundus-first dissection, along with intraoperative cholangiography and alternative approaches like subtotal cholecystectomy.

**Methods:**

This paper introduces the shoeshine technique, a maneuver designed to achieve atraumatic exposure of anatomical structures, local hemostatic control, and ease of infundibulum mobilization. This technique involves the use of a blunt dissection tool and gauze to create traction and enhance visibility in Calot’s triangle, particularly beneficial in cases of severe inflammation. Steps include using the critical view of safety and Rouviere’s sulcus line for orientation, followed by careful dissection and traction with gauze to maintain stability and reduce the risk of instrument slippage.

**Results:**

The technique, routinely used by the authors in over 2000 cases, has shown to enhance patient safety and reduce bile duct injury risks.

**Conclusion:**

The shoeshine technique represents a simple and easy way to apply maneuver that can help surgeon during laparoscopic cholecystectomies exposing the hepatocystic area and promote blunt dissection.

## Introduction

Each year, an estimated 750,000 laparoscopic cholecystectomy procedures is performed in the United States of America, accounting for 90% of all cholecystectomy procedures^1^. Since its introduction in 1985 by Prof. Dr. Erich Mühe from Germany, more than 30 million patients have benefited from this minimally invasive procedure^2^. Consequently, minimally invasive surgery has become the gold standard for treating chronic symptomatic calculous cholecystopathy and acute cholecystitis. It is estimated that, since its introduction, the number of cholecystectomies performed by minimally invasive technique has increased by 10–69% worldwide^3^.

**Figure 1:**
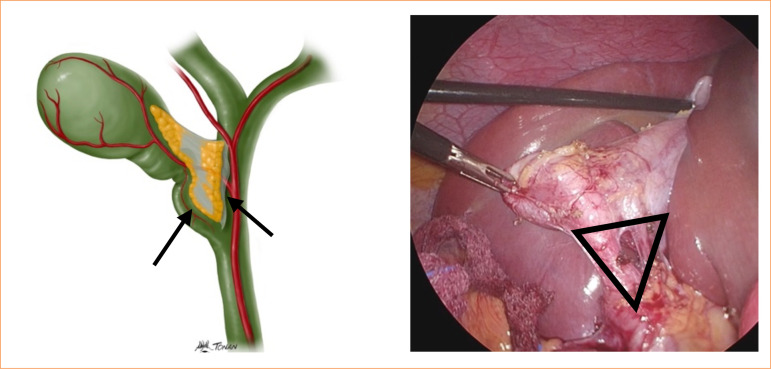
Landmarks of Calot’s triangle (black triangle): the lateral wall of the cystic duct, the central portion of the common hepatic duct (arrows), and the lower edge of the liver.

Source: Elaborated by the authors.

Despite a better understanding of this surgical approach, improved training in minimally invasive surgery, and advances in technology with better cameras, screens, and instruments, the dreaded complication of iatrogenic bile duct injury persists, ranging between 0.2–1.3% of all minimally invasive cholecystectomies^4^. Most bile duct injuries are related to misinterpretation of biliary anatomy, leading to unexpected bile duct injuries. Known risk factors for such complications include male gender, obesity/body habitus, acute cholecystitis, previous hepatobiliary surgery, and anatomical variations or inflammatory changes within Calot’s triangle. This triangle, located between the lateral wall of the cystic duct, the central portion of the common hepatic duct, and the cystic artery, also known as the hepatocystic triangle according to Strasberg and Brunt^5^, presents technical difficulties in accurately identifying the structures, especially in cases of severe inflammation (Fig. 1).

Typically, only the cystic duct and artery are observed in this region^6^. Complications associated with minimally invasive cholecystectomy include bleeding, abdominal infection, damage to surrounding organs such as the duodenum and liver, and bile ducts. It is expected that all surgeons performing this procedure are well informed of the anatomical anomalies of the arteries and the bile ducts to prevent potential significant blood loss and bile duct injury^7^.

Various criteria and technical strategies have been proposed to mitigate the risk of the most dreaded complication associated with cholecystectomy, namely iatrogenic injury, to the common bile duct. One such approach is the critical view of safety, introduced by Strasberg and Brunt^5^ and Strasberg et al.^8^ in 1995. This method emphasizes careful dissection of Calot’s triangle to ensure clear visualization of the structures, most importantly the cystic duct and artery, before proceeding^9^. Another approach introduced recently is fundus-first dissection, which involves starting dissection from the fundus of the gallbladder to the infundibulum in cases in which the structures of Calot’s triangle are difficult to identify^10^.

Additionally, several tips and tricks have been suggested, including the liberal use of intraoperative cholangiography in cases of uncertain anatomy and avoiding excessive cephalad traction on the gallbladder to prevent misidentification of the common bile duct as the cystic duct^11^. In cases in which the dissection of Calot’s triangle is complex, a bailout procedure, such as subtotal cholecystectomy or conversion to open cholecystectomy, should be considered.

Another surgical landmark is Rouviere’s sulcus → segment 4 → umbilical fissure line, aiming to establish a safe zone for dissection and three-dimensional planar considerations to avoid bile duct injury^12^.

This paper aimed to describe a very simple and efficient maneuver named the shoeshine technique. The authors, who have performed this technique in over 2,000 cases, assert its use in achieving atraumatic exposure of anatomical structures, facilitating local hemostatic control, and easing the mobilization of the infundibulum. This maneuver is particularly beneficial in cases of intense inflammation in which conventional grasping forceps may repeatedly slip, complicating the procedure. The maneuver was first presented in Brazil in 2008 during a surgical conference^13^.

## Methods

- Start by locating the gallbladder infundibulum and Calot’s triangle;
- Follow the critical view of safety technique, using the imaginary line from the Rouviere’s sulcus to the umbilical fissure;
- Using a blunt dissection tool, such as a Maryland instrument, carefully open the peritoneum between the infundibulum and cystic duct area until a gap form between the duct and cystic artery (Fig. 2);
  **Figure 2:**
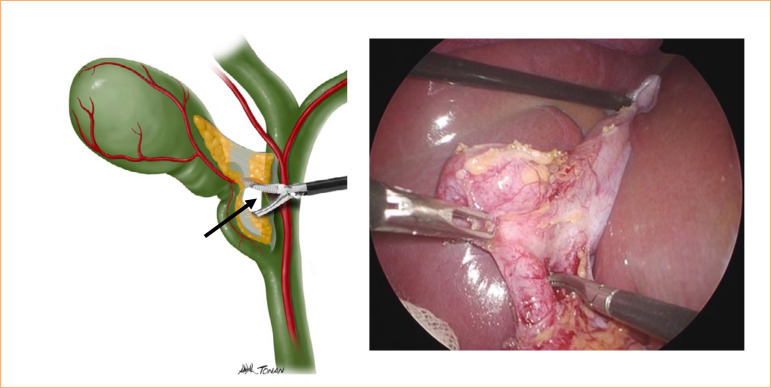
Blunt dissection with Maryland-type instrument, creating a gap between the duct and the cystic artery (arrow).

Source: Elaborated by the authors.
- Introduce a piece of gauze into the gap to aid dissection. Hold the gauze with Maryland forceps and gently maneuver it medially through the region (Fig. 3);
  **Figure 3:**
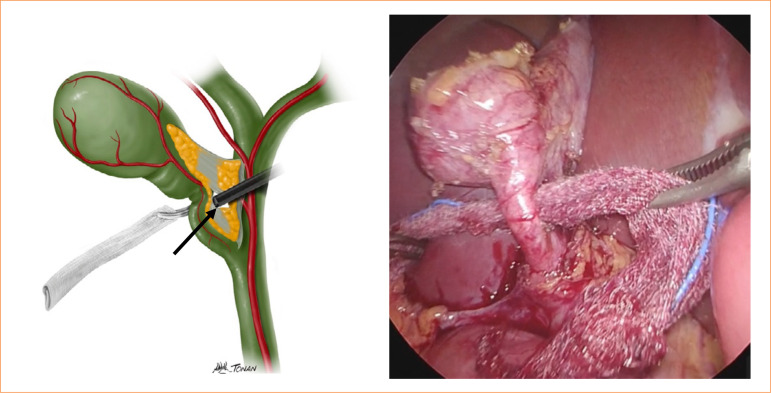
Passing the gauze into the gap to help with infundibular traction.

Source: Elaborated by the authors.
- Grasp both ends of the gauze near the infundibulum to create lateral traction, opening Calot’s triangle;
- Employ gentle lateral and medial movements for blunt dissection, gradually increasing the space between the infundibulum and the common hepatic duct, termed the shoeshine technique;
- This maneuver aids in local hemostasis, particularly helpful in cases with bleeding small branches near the cystic duct, enhancing visibility (Fig. 4);
  **Figure 4:**
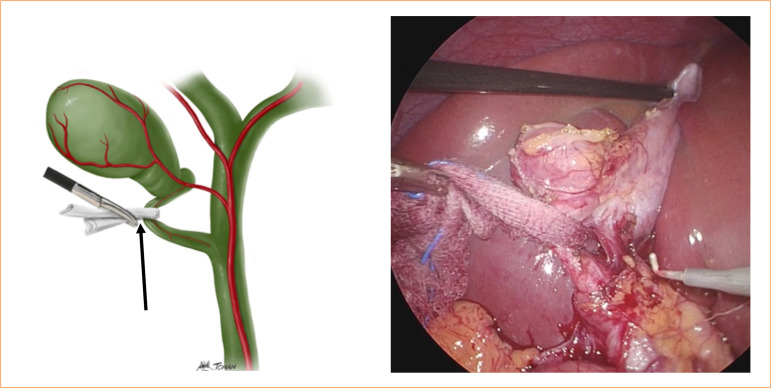
Lateral traction with the gauze helping the mobilization and at the same time providing bleeding control.

Source: Elaborated by the authors.
- By using gauze for traction, the surgeon establishes a stable anatomical space, reducing the risk of instruments slipping, which is common in inflamed tissue, thus facilitating exposure of Calot’s triangle;
- After identifying and treating the cystic artery, remove the gauze and proceed with cholangiography and/or cystic duct ligation as per surgical preference and indication.

## Discussion

Over the past two decades, a variety of technical strategies have been proposed to mitigate the risk of biliary tract injuries during laparoscopic cholecystectomy^9^. A fundamental principle underlying these proposals is the adoption, whenever feasible, of the guidelines delineated by Strasberg in 1995. According to these guidelines, meticulous identification of the cystic duct and artery, along with comprehensive visualization of the liver within the dissected anatomical space, is considered the benchmark for safety^14^.

Additionally, the use of intraoperative cholangiography, though increasingly performed selectively by most surgeons, remains a cornerstone in risk reduction. Furthermore, alternative techniques, such as the top-down approach, partial cholecystectomy, or conversion to an open technique, are recommended in challenging cases. However, it is worth noting that these approaches can pose challenges for inexperienced surgeons, particularly those with limited exposure to traditional procedures^15^.

In the quest to minimize iatrogenic biliary injuries, various techniques have been developed and refined, with intra-operative fluorescent cholangiography emerging as a notable advancement. This technique was first described by Stiles et al.^16^ in 2006 in an experimental study using a mouse model. It involves the administration of indocyanine green, a fluorescent dye that binds to bile and allows for real-time visualization of the biliary anatomy during surgery when exposed to near-infrared light.

In 2018, Pesce et al.^17^ described the importance of using fluorescent vision to help surgeons during laparoscopic cholecystectomies. The procedure involves administering a fluorophore, indocyanine green, which is excreted by the biliary system, and using a near-infrared light source to excite the fluorophore enables visualization of the bile duct system. This enhanced visualization aids surgeons in identifying the biliary structures with greater accuracy, thereby reducing the risk of inadvertent injury. Compared to traditional methods such as preoperative magnetic resonance cholangiopancreatography or intraoperative cholangiography using X-rays, fluorescent cholangiography offers the advantage of dynamic and continuous monitoring without radiation exposure.

In another paper published by Pesce et al.^18^ in 2021, the authors reported that the use of indocyanine green is minimally invasive and can be integrated seamlessly into laparoscopic procedures18. This technique not only improves the identification of anatomical variations, but also facilitates the early detection of bile leaks and other complications, ultimately enhancing patient safety and surgical outcomes. However, one of the major limitations worldwide is its availability and the lack of well-established clinical guidelines.

The technique presented herein introduces a maneuver designed to facilitate traction of the gallbladder infundibulum, particularly in cases in which thickening due to acute inflammatory processes complicates the lateral traction required for achieving the critical view of safety, as described by Strasberg and Brunt5 and Strasberg et al.^8^. Additionally, we have observed that placing gauze across Calot’s triangle aids in blunt dissection of surrounding tissues and helps control bleeding from small vascular branches in the infundibular region, thereby enhancing visualization of anatomical structures. We have adopted this technique for all cholecystectomy cases, noting its simplicity and effectiveness, especially in acute situations.

While this technique has shown significant advantages, its application is limited in cases in which the surgeon cannot confidently identify the space between the cystic duct and the cystic artery. In such situations, a top-down approach is recommended to carefully dissect the gallbladder from the fundus to the infundibulum, followed by intraoperative imaging to accurately identify anatomical structures. Alternatively, partial cholecystectomy may be considered to mitigate the risk of bile duct and right hepatic artery injury^19^.

## Conclusion

The shoeshine technique, meticulously applied by the authors, has proven to be a valuable addition to the surgical repertoire, particularly for cases involving intense inflammation. Its advantages include enhanced traction and visibility in Calot’s triangle, effective local hemostasis, and the establishment of a stable anatomical space, thereby significantly reducing the risk of bile duct injuries. We recommend incorporating this maneuver into laparoscopic cholecystectomy procedures to improve patient outcomes and surgical safety.

## Data Availability

The data will be available upon request.
